# Multiscale architectured materials with composition and grain size gradients manufactured using high-pressure torsion

**DOI:** 10.1038/srep26590

**Published:** 2016-05-27

**Authors:** Ji Yun Kang, Jung Gi Kim, Hyo Wook Park, Hyoung Seop Kim

**Affiliations:** 1 Department of Materials Science and Engineering, Pohang University of Science and Technology, Pohang 37673, Republic of Korea

## Abstract

The concept of multiscale architectured materials is established using composition and grain size gradients. Composition-gradient nanostructured materials are produced from coarse grained interstitial free steels via carburization and high-pressure torsion. Quantitative analyses of the dislocation density using X-ray diffraction and microstructural studies clearly demonstrate the gradients of the dislocation density and grain size. The mechanical properties of the gradient materials are compared with homogeneous nanostructured carbon steel without a composition gradient in an effort to investigate the gradient effect. Based on the above observations, the potential of multiscale architecturing to open a new material property is discussed.

Currently, there is growing interest in the architecturing of materials in the materials science and engineering communities. Architectured materials, which have been defined as combinations of two or more materials or combinations of materials and space in the pioneering papers^1^,^2^,^3^,^4^, open a new possibility to fill gaps in the material property space or Ashby chart^5^, substantial parts of which remain empty. In addition to the expansion of the material property window, architectured materials are also believed to have multi-functional performances^6^ because the variety of feasible combinations of materials and their spatial arrangements theoretically allow them to have numerous possibilities for property control. Moreover, the architecturing of materials indicates a propitious direction to achieve a long-held desire of material scientists for advanced structural materials.

Given this context, the increase in the amount of research on architectured materials is not surprising. Previous studies have established the conceptual frameworks for manufacturing architectured materials and have also strived to demonstrate their experimental feasibility^1^,^2^,^3^,^4^,^5^,^7^,^8^. A wide variety of approaches have been being investigated: gradient structures^9^,^10^,^11^,^12^, diffusion-based architecturing^13^,^14^,^15^, hybrid materials with interlocked structures^16^,^17^,^18^,^19^, and biomimetics of nature’s hierarchical structures^20^,^21^,^22^,^23^,^24^,^25^.

Among these approaches, a combination of the two promising concepts of severe plastic deformation (SPD) and architecturing is regarded as a powerful method to achieve architectured hybrid materials with ultrafine-grained (UFG) and nanocrystalline (NC) structures^8^,^26^,^27^. This approach, or SPD-based architecturing, has significant potential to combat the intrinsic shortcomings of SPD-processed metallic materials, such as the loss of ductility, hardening behavior, and thermal instability, which has functioned as a barrier to their practical application.

A group of researchers who favor this approach introduced viable manufacturing strategies for hybrid architectured materials with a spiral structure using high-pressure torsion (HPT) and helical filament reinforcement using torsion-extrusion^8^. Twist extrusion, which is another SPD processing technique, has also been investigated as a potential method for manufacturing bulk architectured materials with a copper matrix and an aluminum fiber^27^.

Starting from the concept of this SPD-based architecturing, this study goes further to investigate the combination of diffusion-based architecturing and severe plastic deformation (or SPD-processing) of composition-gradient materials. The primary goal of this study is to present a new processing method for manufacturing multiscale architectured materials in an effort to overcome the current boundaries of the material property space. In addition, to the best of our knowledge, the combination of the compositional gradient and SPD processing is reported for the first time; hence, this research is a new and intriguing scientific exploration of architectured materials.

The primary aim of this study is to manufacture multiscale architectured materials via SPD processing of composition-gradient materials and to investigate their microstructure-property relationship. The initial material was an interstitial free (IF) steel. After carbon gradient was introduced by carburization, the material was processed via HPT and then annealed. Discussions on the following three issues are included in the study. First, the effects of high-pressure torsion processing on the microstructure and mechanical properties are investigated. Second, quantitative analyses of the dislocation density variation induced by the composition gradient are performed. Third, the origin of the multiscale microstructure is discussed. Finally, based on these observations, the concept of multiscale architectured materials (MS-ArchiMat) is proposed and its potential to expand the material property space is documented.

## Results

### Microstructural study of the composition-gradient and grain size-gradient structures

**Figure 1:**
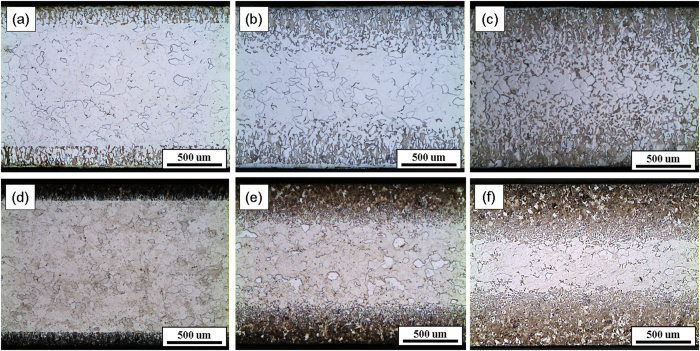
Optical microscopy images of (**a**) C1H-FC, (**b**) C3H-FC, (**c**) C6H-FC, (**d**) C1H-AC, (**e**) C3H-AC, and (**f**) C6H-AC.

**Figure 2:**
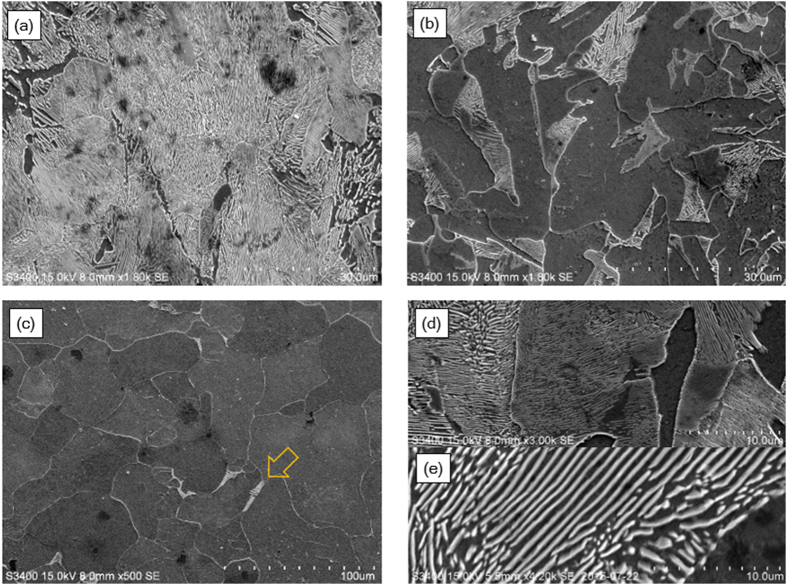
SEM images of (**a**–**c**) C3H-AC and (**d**,**e**) lamella spacing variations of (**d**) C3H-AC and, (**e**) C3H-FC. For C3H-AC, the images are obtained at the different points: (**a**) 50 μm, (**b**) 300 μm from the surface, and (**c**) the center. Pearlite phase was also found in the center region as indicated by an arrow.

The microstructures of the cross-section of the carburized samples observed using OM and SEM exhibited a gradient in the pearlite volume fraction and were also highly contingent on the cooling rate. The OM images in Fig. 1(a–c) represent the furnace-cooled samples, while the microstructures in Fig. 1(d–f) are the air-cooled samples. In addition, the microstructures of C3H-AC are displayed in Fig. 2(a–c), captured at the different points: 50 μm and 300 μm from the surface, and at the center.

Figures 1 and 2 reveal that both the furnace cooled and air-cooled samples in the near-surface region had multi-phase microstructures composed of ferrite and pearlite, but the lamella spacing between the cementite varied in the cooling rate. The coarse lamella bands, which had alternating ferrite and iron carbide layers, with 1.2 ± 0.1 μm distance between them, were observed in the furnace-cooled samples, while the air-cooled samples had very fine lamella structures whose spacing was 300 ± 40 nm, as depicted in Fig. 2(d,e).

It is clear that the carburization depth has an upward trend as the carburizing time increases. In all samples, not only were the carburization depths dependent on the carburizing time but they were also highly affected by the cooling rate. In Fig. 1, the furnace-cooled samples had deeper carburization layers than the air-cooled samples. This result was justifiable because the furnace-cooled samples remained in the furnace for a longer time, which allowed the carbon atoms to have more time to diffuse to the center (i.e. to a low carbon region).

In addition, the carburized samples had a pearlite volume fraction gradient through their thickness. More specifically, the SEM images of C3H-AC in Fig. 2(a–c), which were obtained at different points of 50 μm and 300 μm from the surface, and at the center, demonstrate that the pearlite volume fraction decreased significantly as the observation points became closer to the center. In Fig. 2(a), the microstructure at 50 μm from the surface was almost composed of pearlite, but the pearlite amount decreased in Fig. 2(b), which was observed at 300 μm from the surface. Moreover, the small amount of pearlite phase existed even in the center region of C3H-AC (indicated by the arrow in Fig. 2(c)). This indicates that the carburization depth was far deeper than expected in the OM images.

**Figure 3:**
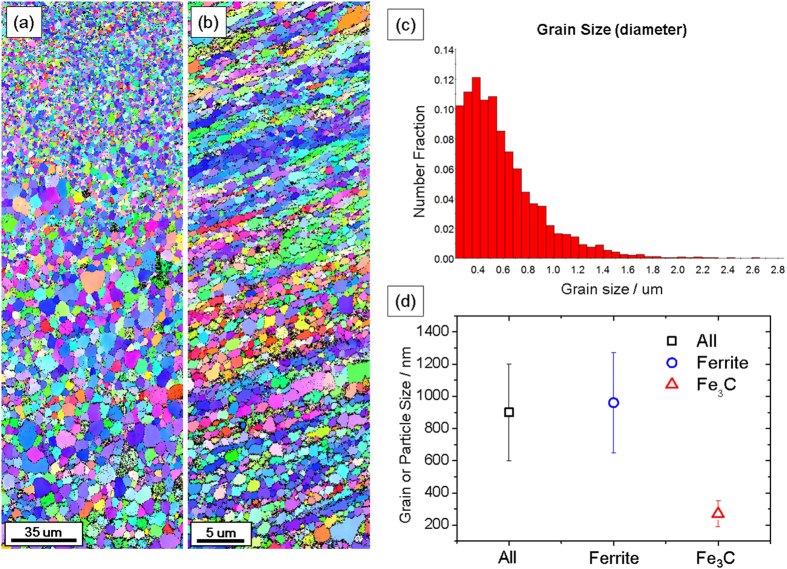
EBSD analyses of the HPT-processed C3H-AC sample after annealing at 650 °C for 10 minutes. Microstructures in the region close to (**a**) the center and (**b**) the surface showed a gradient of grain size. (**c**) The grain size distribution of ferrite grains and (**d**) the average size of the grains and particles in Fig. 3(b) (ferrite grains and iron carbide particles) clearly indicate that they were all in ultrafine-grained regime.

**Figure 4:**
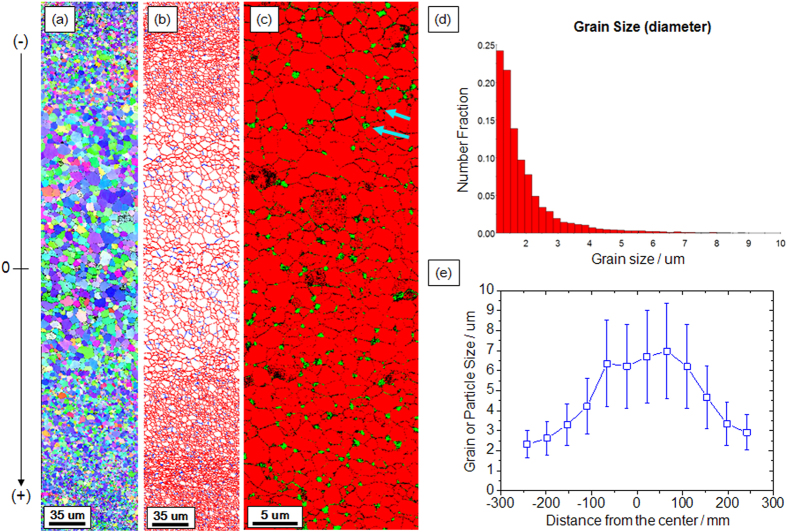
EBSD analyses of the HPT-processed C3H-AC sample after annealing at 650 °C for 2 hours . (**a**) The EBSD image and (**b**) the boundary map clearly representing gradient structure, (**c**) the EBSD phase map with iron carbide precipitates indicated by arrows, (**d**) the grain size distribution of ferrite grains, and (**e**) the calculated average grain size through the thickness.

Figures 3 and 4 display the EBSD images and the calculated grain sizes of the HPT-processed C3H-AC samples that were annealed at 650 °C for 10 and 30 min, respectively. These figures depict the complete decomposition of the cementite phase as well as the multiscale microstructures and ultrafine grains near the surface and coarse grains in the center. The disappearing pearlite phase after the HPT process in this study aligned with the previous research on medium carbon steel with a fine pearlitic structure conducted by Ivanisenko *et al*.^28^,^29^, which indicates that the shear strain corresponding to the five revolutions of HPT in a 10 mm disk was sufficient for the cementite phase to completely dissolve into a non-equilibrium carbon-supersaturated ferrite matrix.

Figure 3(c) presents the size distribution of the near-surface ferrite grains after 10 min annealing at 650 °C and illustrates that most grains were in the ultra-fine grained regime and the number of ferrite grains near 400 nm was the greatest. Thus, the grain size distribution of the specimen in Fig. 3(a) had a multiscale feature. Figure 3(d), which was calculated using the area method, also illustrates that the mean size of all grains and particles (e.g. ferrite and iron carbide) in Fig. 3(b) was 900 ± 300 nm, which remained less than 1 μm. For the iron carbide particles, which reached 270 ± 80 nm, their average grain size was considerably smaller than that of the ferrite grains. It should be noted that, unlike other homogeneous materials whose grains sizes calculated using the area method and number method fall in a similar range, the grain size of the grain size-gradient material in this study calculated using the area method was almost two times larger than that obtained from the number method. This resulted from the large grains being significantly more weighted in the grain size calculation in the area method, although most grains were in the NC/UFG regime.

As the annealing time became longer, the grain growth made the average grain size larger, as the EBSD image and the boundary map indicate in Fig. 4(a,b), respectively. The solid red line indicates high angle grain boundaries (θ > 15°) and the blue line indicates low angle grain boundaries (5° < θ < 15°). It is clearly seen that the grain size gradient remained after 2 h annealing at 650 °C despite the overall grain growth, and most grains were composed of high angle grain boundaries. Moreover, Fig. 4(c), which presents the EBSD phase map of the same material in Fig. 4(a,b) but at a higher magnification near the surface, clearly demonstrates that iron carbide nano-precipitates were primarily formed at the triple junctions of the grain boundaries, as indicted by the arrows, which is in close agreement with the TEM images observed in other studies^28^,^29^,^30^.

### Changes in the strength and ductility after high-pressure torsion

**Figure 5:**
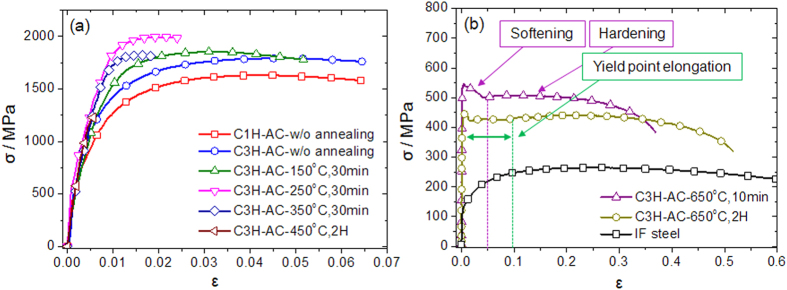
Engineering stress-strain curves of C1H-AC and C3H-AC after HPT and annealing at different temperatures (**a**) less than 450 °C and (**b**) 650 °C. The engineering stress-strain curve of the initial IF steel was plotted together for reference.

Figure 5 depicts the mechanical properties of C1H-AC and C3H-AC after the HPT. They were also demonstrated to vary according to the annealing temperature (T_a_) and time. At T_a_ ≤ 250 °C, the σ_UTS_ of the HPT-processed samples (C3H-AC) increased from 1785 ± 24 MPa to 2007 ± 20 MPa. However, the σ_UTS_ began to decrease significantly at T_a_ ≥ 350 °C and were below 600 MPa at 650 °C, which demonstrated that the decreasing tendency of the tensile strengths accelerated as the annealing time increased. For ductility, the uniform elongation deteriorated steadily. In particular, the materials became considerably brittle at T_a_ ≥ 350 °C, fracturing at a strain of less than 0.01 at 450 °C. Nonetheless, the ductility recovered at higher temperatures near 650 °C to the extent of the initial IF steel.

An unusual plastic feature of the specimens annealed at 650 °C for 10 minutes was the softening and hardening behavior that appeared in the single stress-strain curve. Until 4% strain, the softening behavior dominated the plastic behavior, yet hardening occurred later and continued until the ultimate tensile strength. This aberrant plastic behavior disappeared as the annealing time increased to 2 h and was replaced with a high amount of yield point elongation near 10%.

### Quantification of the average carbon concentration by the element analyzer

The measured average carbon contents of the initial IF steel, C1H-AC, C3H-AC and C6H-AC were 0.0007 ± 0.00008, 0.0276 ± 0.005, 0.0603 ± 0.004 and 0.135 ± 0.006 in wt%, respectively. It is demonstrated that the average carbon concentrations of the carburized samples were in the regime of typical low carbon steel (0.05–0.25 wt%).

### X-ray diffraction analyses

**Figure 6:**
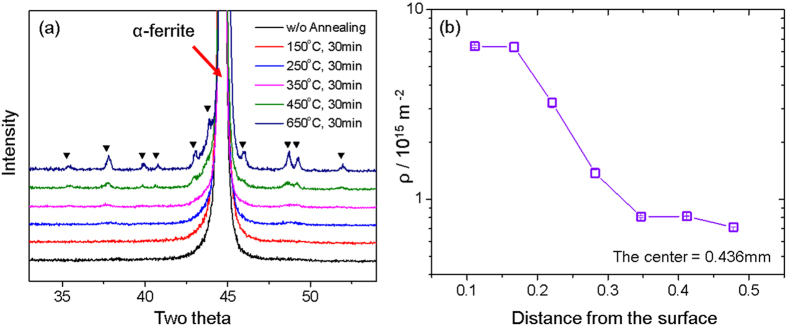
X-ray diffraction analyses: (**a**) the evolution of iron carbide of HPT-processed C6H-AC during annealing for 30 minutes at the different annealing temperatures. XRD measurement were performed approximately at 150 μm at surface. (**b**) Dislocation density (ρ) variation through the thickness direction of the HPT-processed C3H-AC.

The evolution of the iron carbide of the HPT-processed sample (C6H-AC) with different annealing temperatures ranging from 150 °C to 650 °C is presented in Fig. 6(a). After the HPT, the sample without annealing did not exhibit perceptible signs of intensity increases near the Fe_3_C peaks, but the evolution of the Fe_3_C peaks became more distinct as the annealing temperature increased. Through the XRD measurements, the noticeable evolution of carbide peaks was seen above 150 °C.

Figure 6(b) demonstrates that the dislocation density (ρ) had a gradient through the thickness of the HPT-processed C3H-AC sample; the dislocation density near the surface (~10^16 ^m^−2^) was one order higher than that measured around the center (~10^15 ^m^−2^). These were in the range of typical densities for severely deformed materials^31^. Furthermore, the dislocation density appeared to be saturated in two regions: near the surface and at the center.

## Discussion

As previously discussed, the σ_UTS_ of the HPT-processed specimens in this study exhibited a non-monotonic relationship with the annealing temperature (T_a_). This implies that the dominant hardening mechanism varies over T_a_. First, the increasing tendency of the tensile strengths at T_a_ ≤ 250 °C is attributed to the evolution of the carbide nano-precipitates at the defects. The peaks of iron carbide in Fig. 6(a), which began to appear at a low T_a_, implicitly indicate this. Direct evidence has been provided previously by Li *et al*.^32^,^33^ who observed the existence of nanocrystalline carbide precipitates at carbon-segregated subgrain boundaries and triple junctions of heavily cold-drawn hypereutectoid pearlitic steel wires using three-dimensional atom probe and scanning nanobeam TEM diffraction. As T_a_ increases, the boundary migration-induced subgrain coarsening becomes the major microstructural feature that decreases the strength^32^,^33^.

In terms of elongation, on the other hand, the reprecipitation of carbide particles at the defects of nanocrystalline steel seems to be the main factor that negatively affects the ductility. As T_a_ increases to 450 °C and the subgrain coarsening proceeds, subgrain boundary area decreases and therefore, the specific surface area, the total interfacial area between ferrite matrix and precipitates over the total subgrain boundary area, increases. As a result, the boundaries have more brittle feature and become vulnerable to cracks propagation.

Finally, when annealed at 650 °C for 10 and 30 min, the microstructures of the HPT-processed specimens with the carbon gradient were demonstrated to have a multiscale microstructure with an evident grain size gradient (depicted in Figs 3 and 4, respectively). In particular, the stress-strain curve of the specimen annealed at 650 °C for 10 min in Fig. 5(b) presents an intriguing feature: stepwise plastic behaviors of softening and hardening. The engineering stress (σ) in the softening region continuously decreased until ε ~ 0.05 and began to harden after the softening behavior finished. The cause of this phenomenon is clear: multiscale microstructures (depicted in Fig. 3). If it is assumed that all grains in the material are strained to the same extent during the tensile loading (iso-strain condition), higher stress is concentrated in ultrafine-grained regions which have higher dislocation density than in coarse-grained regions. Because high dislocation density leads to low hardening ability, the plastic behavior of the ultrafine-grained region would finish hardening first, i.e. stress saturation, while the coarse-grained center region continued to exhibit hardening. Furthermore, considering that fractures were not found after softening in all samples, this multiscale/gradient microstructure is considered to be resistant to crack propagation after softening which is typically found in severely deformed metals^31^,^34^,^35^.

**Figure 7:**
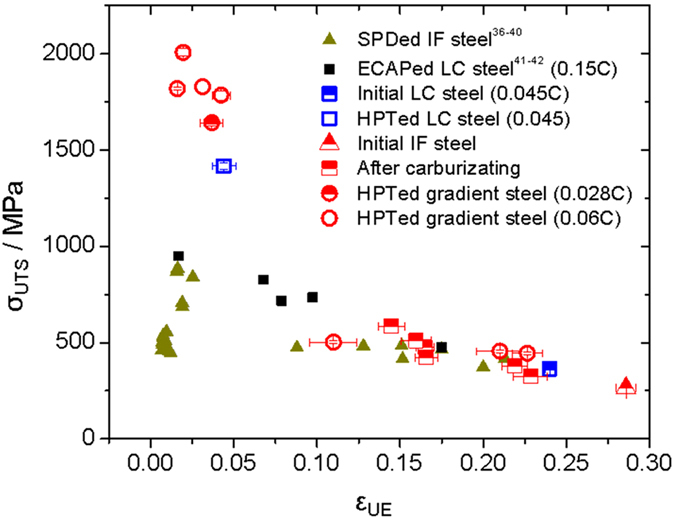
Superior mechanical properties of the materials in the current study compared with those of other SPD-processed interstitial free steel and low carbon steel.

Figure 7 depicts the clear difference between the mechanical properties (σ_UTS_ vs. ε_UE_) of the carbon steel processed by HPT in this work and those of other references^36^,^37^,^38^,^39^,^40^,^41^,^42^. The SPD-processed LC steels that have a comparatively small amount of alloying elements (except carbon) are discussed here for a more thorough investigation of the carbon-gradient effects only. It is evident from Fig. 7 that the gradient materials endure significantly higher stress than the homogenous IF and LC steel counterparts that do not have a gradient structure; the σ_UTS_ are higher and the ε_UE_ remains in the comparable range even though the average carbon concentrations of all samples are similar. (Note that some of the materials in this study have lower amounts of carbon on average than those in other references).

It is considered that the superior strengths of the materials in this study are attributable to the carbon gradient that was introduced. It is widely known that deformation-driven grain refinement is prohibited by dynamic recovery^29^,^43^,^44^. Because subgrain coarsening and dislocation annihilation compete with grain refinement during deformation, the saturation grain size at which the imposed strain does not lead to additional grain refinement, is determined around several hundred nanometers in pure metals^31^. However, the saturation grain size has been known to be significantly smaller in carbon steels at almost one order of magnitude less than that of pure iron^45^,^46^. The underlying reason for this is that carbon segregation to the grain boundaries reduces the driving force for grain coarsening through decreasing the grain boundary energy^43^,^44^,^47^. Based on these results, it is reasonable to think that the material in the present work, i.e. the HPT-processed steel with a carbon gradient, has a gradient in the defects, which is also presented by X-ray analysis in Fig. 6(b).

Related to this, another possible explanation of the improved strengths is the high dislocation density found in the cell walls. The transmission electron microscopy studies of nanostructured pearlitic steels have revealed that dislocations are highly concentrated on the cell walls while very few dislocations exist within the cells^29^,^30^,^48^. These dislocations on the cell walls result in the saturation grain size of the high carbon region in this study being significantly smaller than 100 nm through resisting the dynamic recovery during the constant deformation.

In addition, considering the mean distance between the dislocations and the Hall-Petch law, the gradient effects are more understandable as follows. From Fig. 6(b), it can be inferred that the dislocation density of the high carbon region (ρ_HC_ ~10^16 ^m^−2^ near the surface) was at least one-order of magnitude higher than that of the low carbon region (ρ_LC_ ~10^15 ^m^−2^ near the center). Based on the dislocation density, which was estimated as above, the mean distances between the dislocations, L ~ ρ^−1/2^, of both regions can be calculated: L_HC_ ~ 10 nm and L_HC_ ~ 30 nm. Because the dislocation mean free path is approximately three times higher in the high carbon region than in the low carbon region, the strength of the high carbon region (σ_HC_) is nearly two times higher than that of the low carbon region (σ_LC_) according to the Hall-Petch law (σ ~ L^−1/2^). Therefore, even though the average amounts of carbon in nanostructured steel are similar, the strength of the materials with the composition gradient could be significantly higher, approximately double. This explanation also fits well with the amount of strength increments in Fig. 7.

After annealing, the HPT-processed materials of the carbon gradient had the grain size gradient, as depicted in Figs 3 and 4. The reason for these multiscale features is believed to be the difference in the thermal stability of the surface and center region, which originates from the carbon gradient because the carbon or fragmented carbide particles segregated in the subgrain/cell walls result in the material being resistant not only to dynamic recovery, but also to grain coarsening during annealing^29^,^49^.

All experimental results in this study lead to the possibility of multiscale architecturing to expand the material property space. Here, a conceptual framework for multiscale architecturing is proposed and how it can broaden the material property window is explained.

**Figure 8:**
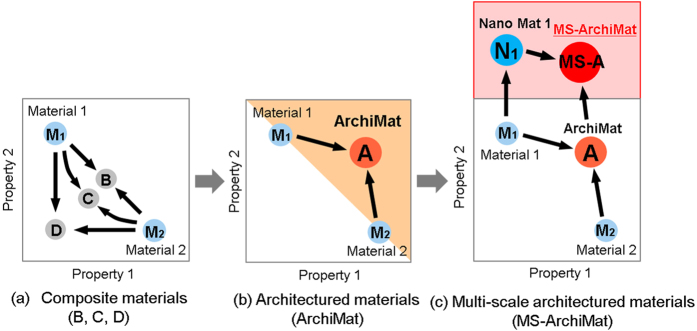
A Conceptual framework of multi-scale architecturing: (**a**) composite materials (B–D) made of two materials (M_1_, M_2_), (**b**) architectured materials (ArchiMat), and (**c**) multi-scale architectured materials (MS-A) made of a nanostructured M_1_ (=N_1_) and M_2_. The colored regions in (**b**,**c**) represent expanded material spaces by architecturing and nanostructuring.

Figure 8(a) presents a possible combination of properties that can be obtained from traditional composite materials made from two conventional materials (M_1_ and M_2_). According to the rule of mixtures, the properties of the final composite material (B, C and D) can be controlled through varying the volume fractions of M_1_ and M_2_. However, the controllable range of properties remains confined to the maximum properties of M_1_ and M_2_. In contrast, the architecturing of the same materials (M_1_ and M_2_) has demonstrated a possibility to enhance properties. The feasible methods that can accomplish this have been reported, such as geometrical hardening^50^, gradient^9^,^10^,^11^,^12^, and interlocked structures^16^,^17^,^18^,^19^, and they provide an avenue for improving the material properties.

In addition to architecturing, an additional method of achieving further improvement in properties is proposed: nanostructuring of the material (from M_1_ to N_1_) and architecturing of the nanostructured material (N_1_) and another material (M_2_). As the schematic in Fig. 8(c) implies, this processing approach to produce multiscale architectured materials (MS-ArchiMat) could significantly expand the material property space even though the materials used are the same as in the other two cases in Fig. 8(a,b).

The advantages of combining architecturing and nanostructuring are evident. First, MS-ArchiMat can exhibit significantly higher specific strength (or strength-to-weight ratio or strength/weight) because the masses are the same for their coarse-grained counterparts. Second, the predictable shortcoming of nanostructured materials could be compensated through architecturing with other materials. Finally, the numerous combinations of architecturing provide an avenue for multi-functional properties. Therefore, multiscale architecturing could be a promising method for designing advanced structural materials.

In summary, the primary goal of this paper was to propose a new direction toward multiscale architecturing. In the current study, a combination of severe plastic deformation and diffusion-based architecturing was proven to manufacture multiscale materials in the following two ways: the gradients in the dislocation mean free path and the grain size. The results demonstrate that among the materials with similar compositions, the gradient material had a significantly higher strength. Despite its potential, this work is limited to the simple one-directional diffusion-based architecturing that does not include unique geometrical effects. In order to truly achieve “three-dimensional multiscale architectured materials (3DMS-ArchiMat)”, it is necessary to introduce other architecturing effects, e.g. hierarchical structures, and it is considered that more vigorous studies on these effects are important for future research.

## Methods

### Carburization

The starting material was IF steel with minimal alloying elements (Fe-0.002C-0.1Mn-0.04Al-0.03Ti-0.01Nb in wt%, received from POSCO). The thickness of the initial IF steel sheet was approximately 1.45 ± 0.05 mm. Solid carburizing was performed in order to introduce the carbon gradient. A stainless steel container was used and carbonous ambience was created through a mixture of approximately 60% graphite powder (mean diameter: 18 μm), 30% BaCO_3_, and 10% NaCO_3_ in the respective volumetric ratio. The IF steel sheets were placed together with the powder mixture inside the container; then, they were completely covered with the powder mixture. Finally, the container was sealed. The carburizing temperature was 900 °C for all samples. There were two variables in the carburization: the carburizing time (1, 3 and 6 h) and the cooling rate (furnace and air cooling). Distinct abbreviations are used for convenience and are summarized as follows: C1H-AC (air-cooled after 1 h carburization), C3H-AC (air-cooled after 3 h carburization), C6H-AC (air-cooled after 6 h carburization), C1H-FC (furnace-cooled after 1 h carburization), C3H-FC (furnace-cooled after 3 h carburization), and C6H-FC (furnace-cooled after 6 h carburization).

### High-pressure torsion process

After carburizing, disk samples with a 10 mm diameter were machined from the sheets and then subjected to HPT under the pressure of 6 GPa with five revolutions. A set of semi-constrained dies was used at a rate of 2 rpm. The average temperature of the disks after the HPT process was 65 ± 5 °C. These HPT-processed samples were annealed at several selected temperatures ranging from 150 °C to 650 °C under an Ar atmosphere in a tube furnace. All samples were cooled in air after annealing.

### Mechanical testing

Plate-type dog bone tensile specimens with a gage length of 1.5 mm were machined from the sheets and disks. For the HPT-processed disks, tensile specimens were obtained at 2.5 mm from the disks’ center. All samples were loaded with a strain rate of 10^−3 ^s^−1^ at room temperature using a universal testing machine (UTM; RB Model 302 Micro Load, R&B Co. Ltd., Daejeon, South Korea). A digital image correlation (DIC) method (ARAMIS v6.1, GOM Optical Measuring Techniques, Braunschweig, Germany) was used to calculate the strain.

In addition, the stress-strain curves of a typical low carbon (LC) steel (Fe-0.0445C-0.26Mn-0.028Si-0.0128P-0.001S-0.068Cu-0.032Ni-0.033Cr) were used for comparison. The 10 mm disks of the LC steel were processed using a three-turn HPT under 6 GPa, which is considered sufficient for full grain refinement. In addition, it should be noted that removing the surface roughness caused by the die asperity decreased the overall thickness of the samples by 80 ± 5 μm.

### Characterization techniques

An optical microscope (OM), scanning electron microscope (SEM; S-3400N, Hitachi Ltd.), and an electron backscatter diffraction (EBSD; Helios, Pegasus, FEI, Oregon, USA) were used for the microstructural characterizations. The samples were etched using 3% Nital etchant after polishing. The EBSD images of the HPT-processed samples were obtained at 2.5 mm from the disk center, and the observations were made perpendicular to the torsion axis. In the EBSD analysis, all points of the confidence index less than 0.09 were eliminated for a reliable microstructural study. It should be noted that the average grain sizes were calculated using an area method^51^, and the grains whose grain size was in the range of 5% to 95% were used for the grain size calculations. For the boundary map, misorientation angles higher than 5° were used.

### Quantification of the average carbon content

Quantifying of the average carbon concentration of the carburized samples is critical to investigating the architecturing effect of the carbon gradient on the mechanical properties. The average amount of carbon in weight percent of the four samples (the initial IF steel, C1H-AC, C3H-AC, and C6H-AC) was measured using an element analyzer (CS 200, LECO Korea Co. Ltd Co.). The detection range of the machine was 4 ppm - 6 wt%, and its precision was 2 ppm or 1% relative standard deviation (RSD). The measurements were performed four times for each sample.

### X-ray diffraction analyses

The X-ray diffraction (XRD) analyses for the HPT-processed specimens between 30° and 55° in two theta (2θ) were performed in order to investigate the evolution of the iron carbide as a function of the annealing time. The peaks of the iron carbide (Fe_3_C) were identified at 35.26, 37.66, 39.83, 40.67, 42.92, 43.78, 45.90, 48.63, 49.16, 51.86 and 54.45 in 2θ, according to PDF card no. 35-0772. The XRD measurements were conducted on the surfaces, which were polished to approximately 150 μm, in the direction parallel to the torsion axis in all samples.

In addition, the quantitative investigation of the dislocation density over the thickness due to the carbon gradient was performed. The surface of C3H-AC after the HPT process was polished, measured using XRD, and then polished again. This process was repeated until the measurement point reached the center of the sample. The distance between the measurements was approximately 60 μm. Line profile analyses of the obtained XRD data were performed using the Convolutional Multiple Whole Profile (CMWP) fitting program^52^ in order to calculate the dislocation density (ρ). The fitting limit was set to 10^−7^, and all fitting parameters were in the error range of less than 5%.

## Additional Information

**How to cite this article**: Kang, J. Y. *et al*. Multiscale architectured materials with composition and grain size gradients manufactured using high-pressure torsion. *Sci. Rep*. **6**, 26590; doi: 10.1038/srep26590 (2016).
